# Performance of a Machine Learning Algorithm on Lesions with a High Preoperative Suspicion of Invasive Melanoma

**DOI:** 10.2340/actadv.v104.40023

**Published:** 2024-07-18

**Authors:** Filippos GIANNOPOULOS, Martin GILLSTEDT, Sofia LINDSKOGEN, John PAOLI, Sam POLESIE

**Affiliations:** 1 Department of Dermatology and Venereology, Institute of Clinical Sciences, Sahlgrenska Academy, University of Gothenburg, Gröna stråket 16, SE-413 45 Gothenburg; 2 Region Västra Götaland, Sahlgrenska University Hospital, Department of Dermatology and Venereology, Gothenburg, Sweden

Preoperative discrimination between invasive and *in situ* melanoma (MIS) is challenging even for experienced dermatologists (1, 2). Moreover, only a few dermoscopic features seem to be useful when differentiating between these 2 entities (3). According to Swedish guidelines (4), a lesion with a high preoperative suspicion of being an invasive melanoma (HPSIM) receives a specific label, which is clearly marked in all patient documentation related to the lesion. The overarching aim with this procedure is to standardize the patient care pathway and shorten the time to surgery. Notably, only lesions suspected of being invasive melanomas receive this label, meaning that this category may be used as a surrogate variable when a dermatologist suspects an invasive melanoma rather than an MIS.

We have previously evaluated the performance of a *de novo* (i.e., model with no pretrained parameters) convolutional neural network (CNN) in discriminating between MIS and invasive melanoma and demonstrated that it performs on par with experienced dermatologists (5–7). Moreover, we have tested how such a model performed on a defined out-of-distribution set consisting of dysplastic nevi (DN) (8). Furthermore, Hernández-Rodríguez et al. recently demonstrated that 2 preconditioned neural networks (i.e., ResNetV2 and EfficientNetB6) outperformed 10 dermatologists in discriminating between thick invasive melanomas (≥ 0.8 mm) and thin invasive melanomas (< 0.8 mm) plus MIS combined (9).

To date, we have only compared dermatologists’ performance in a purely retrospective context without considering the dermatologist’s suspicion in a real-world setting. The aim of this investigation was to analyse how our CNN, which was specifically trained and validated to discriminate between invasive melanoma and MIS, performed on a test set comprising histopathologically verified melanomas (Melanoma test set) and how it performed on a subset of melanomas considered to be invasive preoperatively (HPSIM test set). The secondary aim was to evaluate whether the CNN could distinguish between invasive melanomas within the HPSIM group and invasive melanomas in general and similarly between MIS within the HPSIM group and MIS in general.

## MATERIAL AND METHODS

All dermoscopic images were obtained from the Department of Dermatology at Sahlgrenska University Hospital in Gothenburg, Sweden between 1 January 2016 and 31 December 2022. In this timeframe, most images were obtained using the iPhone 8 Plus (Apple Inc., Cupertino, CA, USA) with a DermLite DL4 dermatoscope (3 Gen Inc., San Juan Capistrano, CA, USA) attached.

Each lesion was represented by a single dermoscopic image. Presence of artifacts like skin markings and hair was permissible. Lesions that were not capturable through a single image and images with inadequate quality were excluded. The original image resolution of the images in both test sets ranged from 1,600x1,200 to 4,416x3,312. All lesions underwent histopathological verification by dermatopathologists.

### Architecture of the convolutional neural network

We used a *de novo* CNN model, with an architecture similar to our previously used model (8). The network consisted of 6 convolutional layers (depths of 16, 32, 64, 128, 128, and 128 kernels) with kernel sizes of 3x3 pixels and a single dense layer (depth 128). A rectified linear unit activation function was used in all layers except the final sigmoid output. Augmentation (transformations including random rotations, scaling, and flips) was used in the training set (Appendices S1–S3). This model achieved a maximum area under the receiver operating characteristic curve (AUC) for the validation set after being trained during 41 epochs for 1 h and 41 min.

### Training of the machine learning model

All available melanoma images from 1 January 2016 to 31 December 2021 (*n* = 1,837) were randomized into a training (*n* = 1,537) and a validation (*n* = 300) set. The proportion of MIS (55%) and invasive melanomas with Breslow thicknesses ≤ 1.0 mm (32%) and > 1.0 mm (13%) was preserved in each set.

The Melanoma test set comprised all eligible dermoscopic images of melanomas histopathologically verified between 1 January and 31 December 2022 (*n* = 476; 169 invasive melanomas and 307 MIS). The HPSIM test set consisted of dermoscopic images of lesions that received the HPSIM label between 1 January and 31 December 2022 (n = 253). Among these, 184 were histopathologically confirmed as melanomas (119 invasive and 65 MIS) (Appendix S4). The other 69 lesions represented other categories, including DN (*n* = 22) (Table SI) (Appendix S5). Only 1 model was evaluated on the test sets. This evaluation was monitored by FG, MG, and SP and the authors verify that only the selected model was evaluated on the 2 test sets.

The study was reviewed and approved by the Regional Ethics Review Board in Gothenburg (approval number 283–18).

### Statistical analysis

All data were analysed using R version 3.5.3 (R Foundation for Statistical Computing, Vienna, Austria). Wilcoxon’s rank sum test was used to compare the sigmoid outputs for different types of lesions and all 2-sample tests. Fisher’s exact test was used to compare proportions. De Long’s test for non-paired samples was used to compare the AUC for the HPSIM and Melanoma test sets, respectively. All tests were 2-sided and *p* < 0.05 was considered as statistically significant.

## RESULTS

There were no significant differences in age and sex distribution between the melanomas in the HPSIM set and the melanoma test set (**Table I**). Also, there was no difference in the lesion distribution on different body sites in the 2 test sets (*p* = 0.22). The mean Breslow thickness was 1.10 mm (95% confidence interval [CI], 0.89–1.32) and 1.13 mm (95% CI, 0.94–1.31) for the HPSIM set and the melanoma test set, respectively (*p* = 0.70).

**Table I:** Receiver operating characteristic demographics

Factor	HPSIM	Melanoma test set
Age, mean (95% CI), SD		
Male	67.1 (64.2–70.0), 15.3	68.0 (66.2–69.8), 14.6
Female	62.4 (58.9–65.8), 14.8	63.5 (61.2–65.8), 16.8
Total	65.2 (62.9–67.4), 16.5	66.0 (64.6–67.4), 15.8
Sex, % (95% CI), *n*		
Male	59.2% (51.8–66.4%), 109	55.5% (50.9–60.0%), 264
Female	40.8% (33.6–48.2%), 75	44.5% (40.0–49.1%), 212

The table displays demographics for the 2 test sets, but only for the melanomas in the HPSIM set. HPSIM: lesions with a high preoperative suspicion of being invasive melanomas.

**Fig. 1:**
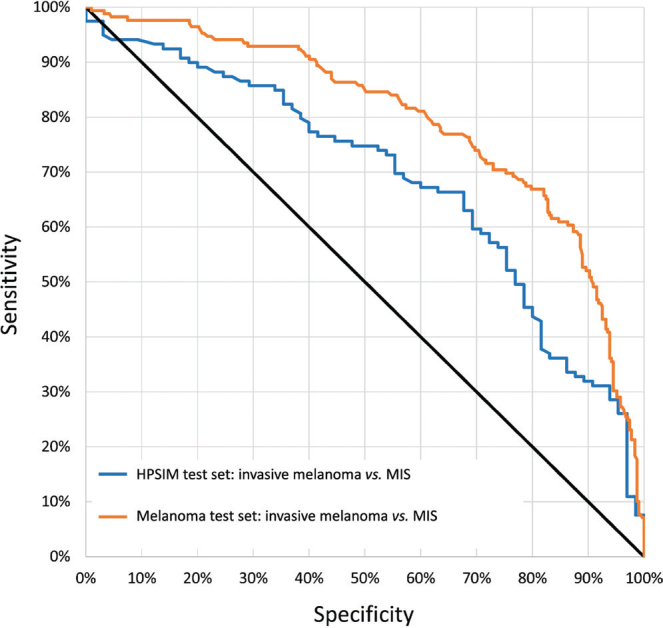
**Receiver operating characteristic curves (ROC).** The figure displays the ROC curves for the 2 respective image sets. The HPSI test set consisted of dermoscopic images of histopathologically confirmed melanomas that received the HPSI label (*n*=184, 119 invasive melanomas and 65 melanoma *in situ*). The All-melanoma test set comprised all dermoscopic images of melanomas with a histopathological verification regardless of the HPSI label (n=476; 169 invasive melanomas and 307 MIS). HPSIM: lesions with a high preoperative suspicion of being invasive melanomas.

**Fig. 2:**
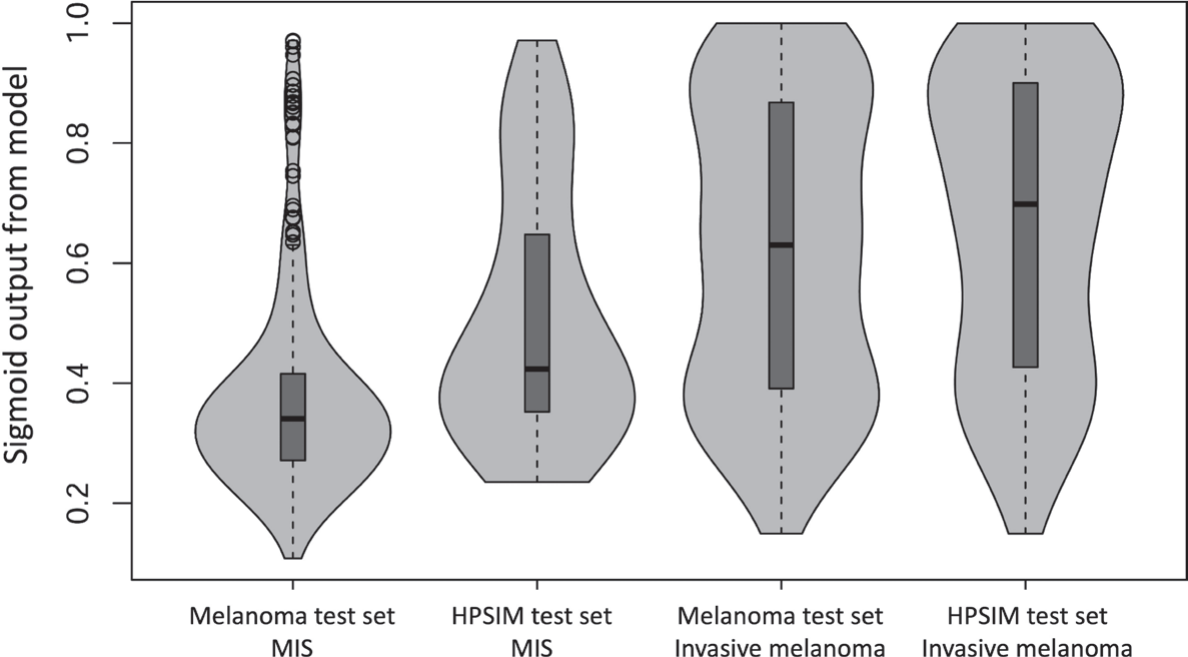
**Violin plots.** The violin plots demonstrate the distinct difference of the sigmoid output between the HPSI melanoma *in situ* and all melanoma *in situ* (MIS) lesions. This illustrates that the HPSI melanoma *in situ* lesions were generally more challenging to diagnose and classify by both the dermatologists and the CNN. CNN: convolutional neural network; HPSIM: lesions with a high preoperative suspicion of being invasive melanomas.

The AUC for the receiver operating characteristic (ROC) (**Fig. 1**) when assessing the CNN’s capability to distinguish between MIS and invasive melanoma in the Melanoma test set was 0.79 (95% CI, 0.75–0.84). The corresponding AUC in the HPSIM test set was significantly lower at 0.68 (95% CI, 0.61–0.76) (*p* = 0.016). When comparing the mean sigmoid outputs for all invasive melanomas in the 2 test sets, there was no significant difference (0.66 vs 0.62, *p* = 0.17). Contrarily, the MIS in the Melanoma test set had a significantly lower score compared with the HPSIM test set (0.38 vs 0.51, *p* < 0.0001) (Fig. 2 and Table SI).

## DISCUSSION

Distinguishing between invasive melanomas and MIS is a frequent and often challenging clinical problem in the preoperative setting. While the CNN evaluated in this study performed well in discriminating between invasive melanomas and MIS in the Melanoma test set, the model exhibited a significantly lower performance for the HPSIM test set. It can be speculated that the primary reason for this is that the MIS lesions in the HPSIM test set typically presented with more “invasive” appearances that were more challenging to evaluate for the dermatologists. It is worth noting that a significant portion of the lesions in the HPSIM test set (53%) were not proven to be invasive melanomas, which emphasizes the complexity of this process as well the evaluation of pigmented skin lesions.

Importantly, this study was not conducted in a prospective setting. However, it still provides insight into how well dermatologists and machine learning algorithms can preoperatively predict which atypical melanocytic lesions have a high likelihood of being invasive melanomas. The implementation of the HPSIM label in Swedish guidelines was designed to ensure prompt and uniform care for patients with a high suspicion of invasive melanoma. However, its efficacy in achieving these objectives vs adding to the healthcare system’s administrative load is a matter for debate. Noteworthily, adherence to our CNN’s outcomes for the lesions in question might have proved beneficial as an adjunctive tool for prioritizing cases, enhancing clinical judgements, and reducing the impact of cognitive biases. Such a practice could potentially reinforce the purpose of the HPSIM label within the Swedish dermatological care framework.

Given the inherent complexities of discrimination between invasive melanomas and MIS, there is a possibility that a more sophisticated and improved version of our algorithm, could be important for applying a wiser choice of surgical excision margins when MIS is suspected, reducing the administrative load, and delivering more precise prognostic information to the patient preoperatively. To this end, our subsequent aim is to launch a clinical trial to evaluate the efficacy of the algorithmic output in a real-world setting.
